# A Review of Antibiotic Efficacy in COVID-19 Control

**DOI:** 10.1155/2023/6687437

**Published:** 2023-10-10

**Authors:** Hamidreza Hekmat, Aziz Rasooli, Zeinab Siami, Kauthar Amir Rutajengwa, Zahra Vahabi, Fatemeh Alsadat Mirzadeh

**Affiliations:** ^1^Cardiology Department, Ziaeian Hospital, School of Medicine, Tehran University of Medical Sciences, Tehran, Iran; ^2^Department of Emergency Medicine, Faculty of Medicine, Tehran University of Medical Sciences, Tehran, Iran; ^3^Department of Infectious Disease, Ziaeian Hospital, School of Medicine, Tehran University of Medical Sciences, Tehran, Iran; ^4^Medical School Department, Ziaeian Hospital, School of Medicine, Tehran University of Medical Sciences, Tehran, Iran; ^5^Geriatric Department, Ziaeian Hospital, Tehran University of Medical Sciences, Tehran, Iran; ^6^Cognitive Neurology and Neuropsychiatry Division, Psychiatry Department, Roozbeh Hospital, Tehran University of Medical Sciences, Tehran, Iran

## Abstract

Severe acute respiratory disease is associated with chronic secondary infections that exacerbate symptoms and mortality. So far, many drugs have been introduced to treat this disease, none of which effectively control the coronavirus. Numerous studies have shown that mitochondria, as the center of cell biogenesis, are vulnerable to drugs, especially antibiotics. Antibiotics were widely prescribed during the early phase of the pandemic. We performed a literature review to assess the reasons, evidence, and practices on the use of antibiotics in coronavirus disease 2019 (COVID-19) in- and outpatients. The current research found widespread usage of antibiotics, mostly in an empirical context, among COVID-19 hospitalized patients. The effectiveness of this approach has not been established. Given the high death rate linked with secondary infections in COVID-19 patients and the developing antimicrobial resistance, further study is urgently needed to identify the most appropriate rationale for antibiotic therapy in these patients.

## 1. Introduction

Coronavirus disease 2019 (COVID-19) is caused by the SARS-CoV-2 coronavirus [1]. To fight the virus, several successful vaccine programs and drug regimes, each with varying degrees of efficacy, have been used. Researchers are increasingly focused on the issue of bacterial illnesses in COVID-19 patients. Serious viral pneumonia is the leading cause of COVID-19 death, particularly among the aged and the frail. Corticosteroids are a type of therapy that has shown the potential in lowering mortality in COVID-19 pneumonia [2]. Immunosuppressive treatment and hospitalization both increase the chance of bacterial infection, albeit. So far, bacterial infections occurred in 8.5% of patients hospitalized with COVID-19 during the first wave of the pandemic [2]. Imagine how difficult it would be to tell the difference between a viral and a bacterial pulmonary illness solely based on clinical appearance. In stark contrast to this, patients with COVID-19 are frequently prescribed medications in general practice [3]. The widespread use of broad-spectrum empirical antibiotic prescribing in COVID-19 may have unexpected consequences, such as an increase in drug-resistant bacteria [4]. We performed a literature analysis with the main aim of evaluating the justifications, supporting data, and practices surrounding antibiotic use in COVID-19 inpatients and outpatients.

## 2. Effect of COVID-19 on the Immune System and the Incidence of Inflammatory

Genetic diversity studies explain that the immune system undergoes major changes in response to the host to various microbial pathogens [5]. At the site of infection in the respiratory tract, some sensor cells such as mast cells, MPS cells, and airway epithelial cells detect opportunistic pathogens. The recognition receptors pattern of these specialized cells is well-equipped with retinoic acid-inducible gene I, Toll-like receptors, and RIG-I-like receptors. As an illustration, double-stranded RNA is also detected by RIG-I generated during coronavirus replication [6]. Despite the body's innate immune barrier against pathogens, coronavirus can affect it. The prominent features of SARS-CoV-2 infection are the suppression immune system and disruption of antiviral type I interferon (IFN).

Furthermore, these viruses can increase mechanisms to control IFN responses in infected cells. Studies have indicated in the first stage, SARS-CoV-2 suppresses the type I IFN of infected hosts by inactivating the IFN regulatory factor III and N proteins as the main viral protein inhibits the expression of IFN [7]. In addition, M proteins repress the generation of type I IFN by preventing the TNF receptor associated factor III protein production. In addition, SARS-CoV-2 accessory proteins include open-reading frame 3b and six proteins act as potent IFN antagonists and oppress the signaling pathway [8]. The expression of IFN-*β* is suppressed by promoting the process of target cell mRNA degradation and inhibiting translation. Infected cells with COVID-19 motivate endoplasmic reticulum protein kinase and the expression of protein kinase R [9, 10]. Generally, based on the mentioned mechanism, reactions of inflammatory are managed by chemokines and inflammatory cytokines like monocyte chemoattractant protein-1, IL-6, and IFN-*γ*-inducible protein-10. In general, clinical evidence has suggested that disease severity is associated with the suppression of IFNs, decreased T and MPS cells, cytokine inflammation, and lung damage [5].

**Figure 1:**
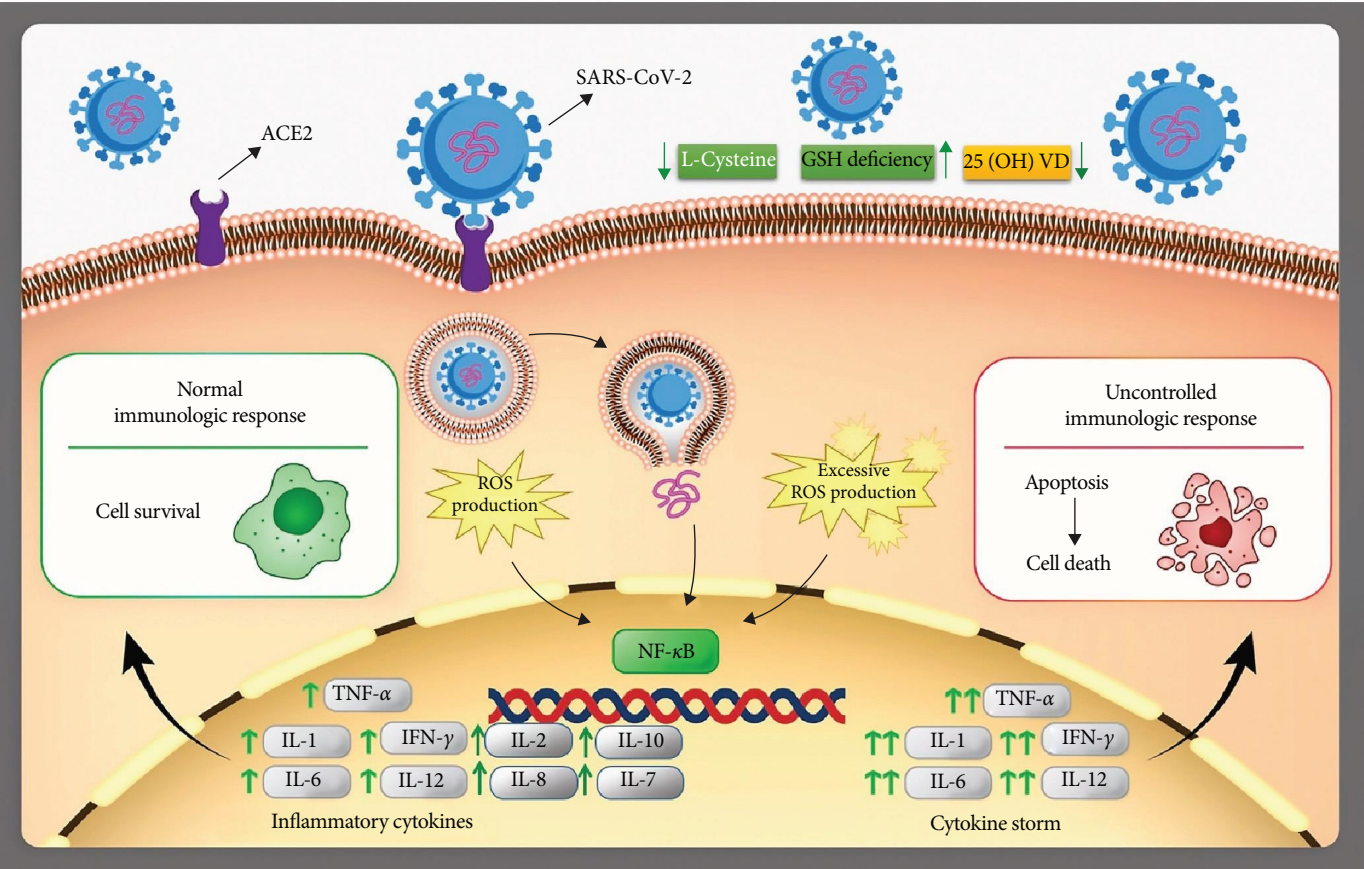
The relationship between SARS-CoV-2 infection and the formation of inflammatory cytokines and the angiotensin-converting enzyme 2 (ACE2) receptor is shown schematically. Cell infection also improves the definition of NF-*κ*B transcription factors that cause the production of the inflammatory cytokines interleukin IL-1, IL-2, IL-6, IL-7, IL-8, IL-10, IL-12, and interferon (IFN); the balance and imbalance in ROS formation [12].

Virus diseases can alter how the NADPH oxidase complex and nitric oxide (NO) synthase are expressed in phagocytic cells, which can alter how reactive oxygen species (ROS) are produced. The creation of cytokines like interleukin (IL), tumor necrosis factors (TNFs), and IFNs is another effect of viral illness (ILs) [11]. As shown in Figure 1, ROS accumulation can cause pathological inflammatory reactions at high levels but is beneficial for immune system function at controlled levels. Humans can produce ROS from up to 1%–3% of the oxygen they breathe in through their lungs, and ROS is crucial for maintaining normal metabolic processes like ATP synthesis. Indirectly or directly, ROS can also level up enzymatic and nonenzymatic antioxidants within the cell, endangering health conditions, and resulting in severe cell harm [12].

Also according to Figure 1, Vitamin D, which has a close relationship with reduced glutathione (GSH), is another important determinant biomarker for both COVID-19 and oxidative stress (OS). It is thought that glutamine reductase (GR) and glutamate cysteine ligase (GCLC) regulate and regulate the production of GSH, respectively [13, 14]. According to the research, vitamin D has the power to modify GCLC and GR on its own. Lack of GSH worsens the disease by increasing OS and the likelihood of extensive protein carbonylation. L-cysteine (LC) has a direct impact on the modulation of protein expression in such a condition, protecting the proteins from the harmful effects of OS [15–17]. Blood sugar levels would rise in situations of OS enhancement, LC intake would increase, and so would the risk for additional infections, including serious COVID-19 infections [17, 18]. On the other hand, a lack of vitamin D has a major role in the development of several chronic illnesses, including insulin resistance, obesity, diabetes, and others. In such circumstances, OS levels rise, GSH declines, and the antioxidant defense system becomes compromised. This cycle continues, and the drawback is that if infection with COVID-19 occurs, tragedy may result [16].

## 3. Control of Coronavirus Infection

Due to numerous genetic alterations, coronaviruses have a high genomic diversity. As a result, it may cross species boundaries and move across species. Nevertheless, no particular and effective antiviral medications have yet been authorized for SARS-CoV-2 infection [19]. Hence, to stop the spread of the disease, preventative measures, and research on viral inactivation are essential. Numerous medications are prescribed for patients and participants in clinical trials to treat SARS-CoV-2 infection depending on clinical needs, including azithromycin (AZ), arbidol, ritonavir/lopinavir, redeliver, chloroquine/hydroxychloroquine, ivermectin, nitazoxanide, minocycline, IFNs, umifenovir, favipiravir, camostat, and tocilizum [8] (Table 1). It is crucial to strike a balance between the rate of risk and efficacy. While treating coronavirus, antibiotics, and particularly AZ, are frequently recommended either alone or in combination with other medications. The amazing thing is that viruses cannot be inhibited by antibiotics; they are only beneficial in preventing and treating microbial illnesses. Due to their inability to completely eradicate the virus, some medications may raise the risk of subsequent infection [34, 35].

Moreover, these drugs can inhibit inflammatory agents only by detecting proinflammatory cytokines and therefore may not be very effective in preventing cytokine storms [30, 36]. For example, AZ is one strong macrolide to modulate macrophage and monocyte responses of cytokine. This antibiotic may balance the immune system mechanism in COVID-19 through declining granulocyte–macrophage acting agent and TNF*α*, IL-6, IL-8, and hindering NF-*κ*B signaling [37].

The oral serine protease inhibitor camostat inhibits trypsin and a variety of inflammatory proteinases, including plasmin, kallikrein, thrombin, and urokinase [27]. It is clinically licensed in Japan as mesylate salt for the treatment of postoperative reflux esophagitis and chronic pancreatitis, but it is not in Europe or the USA due to its ability to inactivate trypsin and impede autodigestion [38]. When used in conjunction with other cell-surface proteases that are involved in activating SARS-CoV-2, camostat mesylate has been demonstrated to have inhibitory effects [39]. In fact, since it was demonstrated that TMPRSS2 had a significant role in the viral pathogenesis of monobasic H1N1, H3N2, and H7N9 influenza A viruses [40], it was identified as a potential target for antiviral drugs.

Nitazoxanide, a structural analog of niclosamide, is an antiprotozoal drug with antiviral activity. It is hydrolyzed to form its desacetyl derivative, tizoxanide. MERS-CoV replication in LLC-MK2 cells is inhibited by nitazoxanide, as is SARS-CoV-2 replication in Vero E6 cells. The antiviral mechanism of tizoxanide is similar to that of niclosamide, as observed in RAW264.7 cells treated with it. It is proposed to conduct a placebo-controlled study of nitazoxanide as postexposure prophylaxis for COVID-19 older individuals and healthcare workers. In other clinical studies, nitazoxanide will be combined with hydroxychloroquine or ivermectin.

An artificial intelligence algorithm identified members of the numb-associated kinases (NAK) family as potential therapeutic targets against SARS-CoV-2 [41]. The AP2-associated protein kinase 1 (AAK1) is a NAK member that binds to clathrin and phosphorylates the medium subunit of AP2, controlling clathrin-mediated endocytosis [42], and its inhibition has been demonstrated to lower the infectivity of a number of viruses [41]. Chemically related pyrazolepyrolopyrimidine derivatives baricitinib and ruxolitinib are clinically approved Janus kinase inhibitors with significant inhibitory effects on AAK1, and are also expected to reduce SARS-CoV-2 infection [43].

In general, the announced drugs do not have antiviral effects, and only studies have been performed on their impact on secondary infections or strengthening the immune system of patients [19].

## 4. Side Effects of Antibiotics Administration in Hospitalized COVID-19 Patients

The adverse impact of taking antibiotics can also cause diarrhea, which is one of the signs of COVID-19. Maslennikov et al. [44] carried out a cohort study to characterize the various types of gastroenteritis that COVID-19 patients encountered. In this cohort analysis, 161 (16.7%) patients had late antibiotic-associated diarrhea, while 89 (9.3%) patients had early viral diarrhea (731 patients had no diarrhea). A amount of 70.5% of those examined who had late diarrhea and none who had early diarrhea had *Clostridioides difficile* infection. Late defecation was associated with a greater risk of dying after 20 days of the disease (*p*=0.009). Notably, the use of oral clarithromycin and amoxicillin/clavulanate (OR: 3.79) increased the chance of the development of late diarrhea.

An observational, retrospective, multinational, 1 : 3 case–control study was carried out on COVID-19 patients to learn the incidence, prognoses, and risk factors for *Clostridioides difficile* infections [45]. The 8,402 COVID-19 patients who were admitted to eight Italian institutions for this study were discovered to have 32 hospital-onset *Clostridioides difficile* illnesses. There were 4.4 hospital-onset *Clostridioides difficile* infections per 10,000 patient days overall. The average duration of inpatient admissions for patients was 35.0 versus 19.4 days (*p*=0.0007). The finding that the use of medicines during the hospital stay was a risk factor associated with the onset of *Clostridioides difficile* infection in COVID-19 patients (*p*=0.004) is noteworthy.

## 5. Antibiotics Azithromycin and Secondary Infections

AZ is an antibiotic of broad-spectrum macrolide with a long half-life (almost 68 hr) and a wide range of broadcasts [46]. AZ is a 15-membered ring azalide (C_36_H_72_N_2_O_12_. H_2_O; *M*_*r*_ 785.02; CAS 83905-01-5), which was synthesized from erythromycin in early 1980. It has been altered to have better tissue infiltration, improved resistance to acid decay, and lesser gastrointestinal side effects [47]. Like other macrolide antibiotics, AZ accumulates in cells, especially phagocytes, thereby inhibiting bacterial protein synthesis and biofilm formation. AZ acts as an inhibitor in bacteria by binding with the 50S large ribosomal subunit and the growth of the polypeptide chain [20]. Due to such a mechanism of action, AZ was proposed to treat a range of Gram-positive and Gram-negative bacteria. The basicity of AZ leads to a swift entrance of the outer membranes and more effective penetration into the bacteria [48]. AZ is effective against Gram-negative organisms such as *Haemophilus influenza*, *Moraxella catarrhalis*, *Streptococcus* spp., and *Staphylococcus* spp. as Gram-positive bacteria. Moreover, this antibiotic has activity against some *Enterobacteriaceae* such as *Escherichia coli*, *Enterobacter cloacae*, and *Shigella* species.

Other studies demonstrate that AZ affects bacteria like *Legionella pneumophila*, *Borrelia burgdorferi*, *Mycoplasma pneumonia*, *Mycobacterium avium*-intracellulare, *Ureaplasma urealyticum*, *Chlamydia trachomatis*, and some protozoa like Cryptosporidium and Plasmodium species [5, 49–56] (Table 2). It is, therefore, on the WHO list of essential medications and has been manufactured in a large scale globally [57]. The results of clinical trials during virus infections such as influenza, SARS-CoV-2, and COVID-19 proved secondary infection with a high percentage of bacterial infection in patients [5, 58]. Almost less than 3 *μ*M of AZ is adequate to deter bacterial pathogens in respiratory diseases such as bronchitis and pneumonia. There is no random clinical confirmation to support the efficacy of AZ as an antiviral and therapist for COVID-19 patients other than coinfection conditions [35]. In China, antibacterial antibiotics such as AZ, fluoroquinolones, or amoxicillin have been used only for patients with mild symptoms of bacterial infection [59]. Most antibiotic-related treatments are commonly applied for coinfection in China and Italy [34]. In general, antibiotics are prescribed for more than 75% of COVID-19 patients admitted to hospitals. This is probably aimed at treating secondary bacterial infections, not their effect on modulating the immune system or controlling the virus [46, 60].

## 6. Interaction between Mitochondria, OS, and Inflammation in COVID-19 Infection

OS is associated with antioxidant and oxidant (species of reactive nitrogen/oxygen) and system imbalances involving the cellular damage process. This disorder could result from a loss of antioxidant capacity or an excessive quantity of ROS. ROS as by-products are produced by different enzymes in peroxisome compartments, mitochondria, and endoplasmic reticulum [61]. The superoxide anion and its derivatives hydrogen peroxide and hydroxyl radical are the main active chemical species containing oxygen. Although ROS are essential for some cellular acts as activation of transcription factors, gene expression, and protein phosphorylation, their uncontrolled production leads to an indiscriminate oxidative attack on inflammation response, proteins, lipids, cell death, and organ damage [62, 63]. Many studies have been performed on the effects of free radicals and OS on organs such as the liver, kidneys, and cardiovascular, all of which confirm the negative and destructive effects of OS on body tissues and organs. Some of these investigations show that oxidative damage, oxidizing agents such as aflatoxin B1, and sodium nitrate lead to damage to various organs such as the liver. In these studies, thymoquinone, Allium Tirtifolium Boiss (Persian shallot) extract, and alcoholic extracts of watercress reduced the formation of free radicals, oxidation of liver proteins, hydroxyproline content, and increased glutathione peroxidase activity. In addition, metformin (250 and 500 mg/kg) as an antioxidant and protective plays a crucial role in reducing OS, plasma iron, and lowering glutathione levels in antioxidant effects on liver fibrosis [64–67]. In addition, OS plays an essential role in the pathogenesis of ischemia-reperfusion injury and consequently renal dysfunction. The use of vitamin E has a protective role against kidney damage by reducing chromium and urea and increasing the glomerulus filter [68]. Vitamin E and ethanolic extract of *Nasturtium officinale* positively protect the lungs against pulmonary fibrosis as a potential antifibrotic factor by reducing ROS destructive efficacy, collagen accumulation, and immune defects [69]. Another study investigating the effects of arsenic trioxide on the oxidative degradation of multiple unsaturated acids in cardiac cell membranes found pentoxifylline and other thiol-based antioxidants (as catalysts in the disulfide exchange reaction) help scavenge free radicals and prevent heart damage during stress [70]. Mitochondria are the well-known leading source of ROS in cells and are crucial for balancing ROS production and scavenging for the optimal functioning of cells [71].

Viral infections can inhibit various mitochondrial functions and thus harm the production of free radicals. The formation of free radicals is associated with the release of mtDNA into the cytosol [72–74]. During the interaction of the virus with the host cells, ROS can be generated by biotransformer enzymes such as spermine oxidase, cytochrome P450, and xanthine oxidase. However, it seems that increased OS could be used as a survival and resistance strategy for viruses [75]. Clinical research confirms that the intensity of free radicals can induce inflammation and cytokines like TNF-*α*, IL-1*β*, and IL-6. These cytokines are salient results of COVID-19 disease severity. They deter mitochondrial oxidative phosphorylation, ATP generation and start ROS production in the cells of the host. In addition, inflammatory mediators and immune sentinels drive intracellular cascades that change the metabolism of mitochondrial. Mitochondrial dysfunction and impaired inflammation of the immune system have a reciprocal effect on each other [76].

## 7. Antibiotics and Mitochondrial Targeting

**Figure 2:**
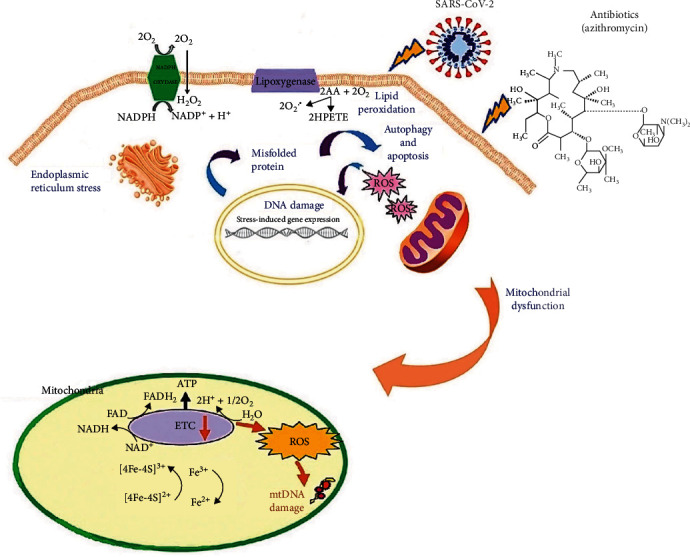
Antibiotics like azithromycin and the SARS-CoV-2 virus are shown in a schematic as the stimuli that cause the production of reactive oxygen species (ROS) and the paths that lead to mitochondrial dysfunction, which cause cell damage [87].

According to studies on secondary infection statistics, antibiotic prescription rates (94%–100%) are greater than the reported secondary infection rates (10%–15%). In truth, antibiotics are frequently used to treat the coronavirus in addition to secondary illnesses. However, the efficacy of antibiotics against the virus has not yet been established, and using them carelessly in situations where there are no secondary illnesses is linked to adverse effects and mitochondrial malfunction [77–79]. The virus is constantly mutating, and geographical regional differences in the structure of the coronavirus lead to differences in the type and extent of infection, transmissibility, and consequently the severity of COVID-19 disease. As a result, prescribing a specific range of medications to all coronavirus patients will not have the same effect and is not recommended. It is clear that bacteria are the primary target of antibiotics to reduce infection [35]. Unfortunately, if antibiotics are used for patients without symptoms of bacterial infection and repeated use, they can target mitochondria that are evolutionarily and genetically related to bacteria [80]. Observations by Zhang et al. [81] and Jiang et al. [82] showed that AZ also caused mitochondrial toxicity, overproduction of ROS, and oxidative DNA damage in patients with mild symptoms. They also suggested that side effects of antibiotics may be a major factor affecting patients' immune responses and overall bioenergy during viral infections [81–83]. In general, antibiotics not only do target bacteria but also induce mitochondrial damage. Inhibition of biogenesis and mitochondrial activity leads to impaired ATP production, metabolism, and ultimately cell death [84]. Antibiotics used to manage COVID-19 in Wuhan have been shown to have an inhibitory effect on mtDNA synthesis [85, 86].  Figure 2 details cellular disorders and mitochondrial mechanisms under the influence of coronavirus and antibiotics. Under normal conditions, electrons in a stepwise fashion transfer through-composed of respiratory chain complexes (ETC) I–IV until they finally downturn O_2_ to form H_2_O_2_. The generated O^2−^ (from complex I of mitochondria) is eliminated by mitochondrial manganese superoxide dismutase to produce water (from complex III of mitochondria) [87–89]. Apoptosis has been identified as a significant mechanism of NP-induced OS-induced cell death [90, 91]. Because mitochondria are one of the key target organelles for NP-induced OS, the intrinsic mitochondrial apoptotic pathway plays a significant role in metal oxide NP-induced cell death [87, 92]. High amounts of ROS in the mitochondria can cause membrane phospholipid damage and depolarization of the mitochondrial membrane. While low or medium ROS levels stimulate mitogenic signaling via reversible oxidations, high ROS levels cause nucleic acid and lipid oxidation and peroxidation, which results in cellular death and necrosis [93].

Electrons are transferred to bearer molecules such as FAD+ and NAD+ and generate NADH and FADH2. Under normal conditions, protons are transferred from the matrix to the intermembrane space of the mitochondria through the energy from the electrochemical gradient of ATP conduction and electron transfer in ETC. However, all antibacterial drugs as toxic agents use internal iron released from iron–sulfur clusters to elevate Fenton-mediated hydroxyl radical formation by the tricarboxylic acid cycle and depletion of NADH. Antibiotics lead to an unbalance between ROS mechanisms and cellular antioxidant protection. As an explicit example, highly reactive ROS, including peroxynitrite (ONOO^−^) and hydroxide (OH) produce from reacting between O^2−^ and H_2_O_2_ with NO and ferrous ion (Fe^2+^), respectively. Furthermore, these ROS have detrimental cellular effects on the oxidation of proteins and lipids, mitochondrial membrane integrity, function of ETC complexes, and mtDNA damage [94–96]. Antibiotics and other groups of drugs inhibit the ETC and its complex respiratory enzyme activities. Hinder of ETC leads to rising ROS formation, ATP production disorder, and mtDNA replication. Damage to mtDNA leads to impairment of the translation system and then malformed synthesis of mitochondrial proteins, which subsequently increases the amount of ROS production [97]. Hence, the accumulation of ROS causes OS and activation of apoptotic pathways in mitochondria and cell death [98]. Destructive factors induce membrane permeability transition pores (mPTP) by inducing structural changes in these membrane proteins. Therefore, overloaded calcium and OS occur. Finally, the result of the inhibition of ETC enzyme complexes or formation of mPTP is depletion of ATP and apoptosis-inducing factor (AIF). The mitochondrial proteins and AIF are released into the cytosol of cells through mPTP, enter the nucleus, and promote nuclear DNA fragmentation causing programmed cell death [99, 100]. Moullan et al. [101] have shown that tetracyclines disrupt mitochondrial function by creating imbalances in the dynamics and proteins of this organelle. In addition, they found that the fatality of the disease was more pronounced in the elderly [77, 102]. This is due to the association of age with decreased mitochondrial biogenesis, increased mutations in mtDNA, and increased mitochondrial ROS levels. Therefore, the use of therapies aimed at improving mitochondrial activity and reducing antibiotic use may be particularly useful in the treatment of COVID-19 in the highest-risk group (over 65 years of age) [34].

## 8. Conclusion and Perspective

The use of antibiotics in COVID-19 inpatients and outpatients was evaluated based on the data that was at hand. Data indicated that superinfections during treatment are rare and that bacterial coinfection at the time of diagnosis is comparatively unusual. The included studies, however, were diverse in that they were carried out in various contexts and adhered to different antibiotic stewardship principles and infection prevention and control protocols. Furthermore, there were significant variations in the methods used to identify individuals who acquired bacterial infections; some studies provided little detail on the techniques employed. In spite of these drawbacks, the discovery allays worries that the epidemic might result in a worrying rise in the frequency of bacterial illnesses.

To support the preliminary result, additional research is required. Antibiotic resistance and adverse effects like *Clostridioides difficile* infection could be managed by reducing the present misuse of antibiotics. When treating COVID-19 patients who fall into high-risk categories, such as the aged, hematologic patients, those getting immunosuppression after solid organ donation, and those whose humoral immunity has been compromised, antibiotic use should be taken into account. According to WHO recommendations, empiric antibiotics should be used to treat all probable pathogens as soon as feasible, and empiric antibiotic therapy should be taken into consideration for potential pneumonia. A dedicated prediction model of bacterial infection in hospitalized COVID-19 patients could help identify subgroups of patients who should receive empirical antibiotic treatment because the prevalence of bacterial coinfection and superinfection during SARS-CoV-2 infection is different from other pandemics. Unless there is a clear clinical suspicion of a bacterial superinfection, antibiotics should not be given at home.

## Figures and Tables

**Table 1:** List of drugs which is declared for clinical trials against SARS-CoV-2.

Drugs	Performance and effect	Direct effect on the SARS-CoV-2 and secondary infection	Reference
Azithromycin	Inhibits bacteria by binding and interfering with the assembly of the 50S large ribosomal subunit	Secondary infection	[20]

Lopinavir/ritonavir	Antiviral (both RNA and DNA genome)	Direct effect	[21]

Hydroxychloroquine/chloroquine	Disrupts in glycosylation of cellular. Increased pH level in lysosomes, disruption of viral receptors for binding to host cells	Secondary infection	[22]

Remdesivir	Inhibits RNA-dependent RNA polymerase. Causing premature termination of viral RNA chains	Direct effect	[23]

Ivermectin	Inhibits the replication of viruses	Direct effect	[24]

Favipiravir	Inhibits viral RNA polymerase and capping of mRNA	Direct effect	[25]

Umifenovir	(Both RNA and DNA genome)	Direct effect	[26]

Camostat	Inhibit of the enzyme transmembrane protease, serine 2 (TMPRSS2)	Direct effect	[27]

Nitazoxanide	Inhibits the protease	Direct effect	[28]

Minocycline	Reduces the cytokine production	Secondary infection	[29]

Corticosteroids	Anti-inflammatory	Secondary infection	[30]
humanized monoclonal antibody (tocilizumab, sarilumab, adalimumab, and siltuximab)	Blocks soluble and membrane-bound IL-6 signal transduction	Secondary infection	[31]

Interferons	Inhibits many stages of virus replication	Secondary infection	[32]

Oseltamivir	Inhibits neuraminidase	Direct effect	[31]

Arbidol	Inhibits membrane fusion	Direct effect	[33]

Methylprednisolone	Anti-inflammatory	Secondary infection	[8]

Baricitinib/ruxolitinib	Inhibits Janus kinase	Secondary infection	[31]

**Table 2:** List of bacteria involved in coinfection with COVID-19.

Bacteria	Effect and infection	Reference
*Haemophilus influenza*	Exacerbation of symptoms and increased hospital stay in the intensive care unit	[5]
*Moraxella catarrhalis*	Effective immune response disruptor and pathogenic synergistic agent	[5]
*Streptococcus pneumonia*	Not completely defined	[49]
*Staphylococcus aureus*	Necrotizing pneumonia	[50]
*Enterobacter cloacae*	Pneumonia	[51]
*Escherichia coli*	Pneumonia	[52]
*Shigella* spp.	Pneumonia	[53]
*Legionella pneumophila*	Pneumonia	[54]
*Borrelia burgdorferi*	Pneumonia	[55]
*Mycobacterium avium*-intracellulare	Interstitial pneumonia	[5]
*Chlamydia trachomatis*	Pneumonia	[5]
*Ureaplasma urealyticum*	Pneumonia	[56]

## Data Availability

Data supporting this research article are available from the corresponding author or first author on reasonable request.
